# The influence of emotional interference on cognitive control: A meta-analysis of neuroimaging studies using the emotional Stroop task

**DOI:** 10.1038/s41598-017-02266-2

**Published:** 2017-05-18

**Authors:** Sensen Song, Anna Zilverstand, Hongwen Song, Federico d’Oleire Uquillas, Yongming Wang, Chao Xie, Li Cheng, Zhiling Zou

**Affiliations:** 1 Faculty of Psychology, Southwest University, Chongqing, 400715 China; 2 Department of Psychiatry, Icahn School of Medicine at Mount Sinai, New York, NY 10029 USA; 3 School of Humanities and Social Science, University of Science and Technology of China, Hefei, 230026 China; 4 Department of Neurology, Massachusetts General Hospital, Harvard Medical School, Boston, MA 02114 USA; 5 Sino-Danish College, University of Chinese Academy of Sciences, Beijing, China; 6 Faculty of Education, Beijing Normal University, Beijing, 100875 China

## Abstract

The neural correlates underlying the influence of emotional interference on cognitive control remain a topic of discussion. Here, we assessed 16 neuroimaging studies that used an emotional Stroop task and that reported a significant interaction effect between emotion (stimulus type) and cognitive conflict. There were a total of 330 participants, equaling 132 foci for an activation likelihood estimation (ALE) analysis. Results revealed consistent brain activation patterns related to emotionally-salient stimuli (as compared to emotionally-neutral trials) during cognitive conflict trials [incongruent trials (with task-irrelevant information interfering), versus congruent/baseline trials (less disturbance from task-irrelevant information)], that span the lateral prefrontal cortex (dorsolateral prefrontal cortex and inferior frontal gyrus), the medial prefrontal cortex, and the dorsal anterior cingulate cortex. Comparing mild emotional interference trials (without semantic conflict) versus intense emotional interference trials (with semantic conflict), revealed that while concurrent activation in similar brain regions as mentioned above was found for intense emotional interference trials, activation for mild emotional interference trials was only found in the precentral/postcentral gyrus. These data provide evidence for the potential neural mechanisms underlying emotional interference on cognitive control, and further elucidate an important distinction in brain activation patterns for different levels of emotional conflict across emotional Stroop tasks.

## Introduction

Cognitive control is the ability to arrange mind and action in line with task-related goals, and consists of a variety of distinct executive processes that include attention shifting, error monitoring, maintenance, and updating of working memory, and reaction conflict or inhibition^1, 2^. Cognitive conflict, within the context of cognitive control, occurs when processing of task-relevant information is challenged with a potent distractor^3^, such as emotionally-salient stimuli that may potentially signal danger (i.e., emotional interference)^4, 5^. Cognitive conflict can arise from this ‘emotional interference’, and can compromise the ability to complete tasks requiring cognitive control^6^. To complete our daily work and study however, efficient emotional interference resolution is crucial.

Rather than viewing emotion and cognitive control as brain functions that operate independently, numerous studies have recently suggested a shared neural circuitry underlying cognitive-emotional conflict resolution^7–9^. For example, there is compelling evidence that brain regions commonly associated with cognitive control, such as the dorsolateral prefrontal cortex (DLPFC), also play an important role in emotion processing^10^. However, thus far, no consensus has been reached as to which neural mechanisms may specifically underlie emotional interference on cognitive control (i.e., the monitoring and resolution of this conflict).

A recent meta-analysis of 43 studies encompassing different tasks in which emotion was intermixed with a variety of “classic” cognitive control tasks (e.g., Stroop, n-back, stop signal or the go/no-go task)^11^, explored the neural mechanisms of the interaction between cognition and emotion, and showed consistent brain activation in both cognitive control [e.g., dorsolateral prefrontal cortex (DLPFC), and inferior frontal gyrus (IFG)] and emotion processing [e.g., subgenual anterior cingulate cortex (ACC) and amygdala regions]. However, the different tasks included in that meta-analysis measure different psychological processes. For example, the n-back task was included to study working memory processes^2^, while the go/no-go and stop signal task investigate response inhibition^12^, and both the Stroop and Flanker paradigms study conflict resolution^3^. Furthermore, although the Stroop and Flanker task were the two main paradigms exploring cognitive conflict in that meta-analysis, there are important differences even between these two tasks. For example, emotional Flanker tasks emphasize conflict resolution as relevant to spatial location, requiring a subject to attend to a centrally-fixated stimulus while ignoring flanking stimuli^13–15^. The emotional Stroop task, on the other hand, focuses on color-semantic or number-semantic conflict^16, 17^. Therefore, here we focused specifically on the emotional Stroop task, hoping to reveal the neural mechanisms underlying emotional interference on cognitive control.

The emotional Stroop task is frequently used in fMRI studies for exploring the neural mechanisms of the interaction between emotion and cognition^6, 18^. A study by Mohanty and colleagues (2005) was one of the first studies to examine the changes in brain function during an emotional Stroop task^19^. They showed significantly increased brain activation for negative words versus neutral words, in the IFG, ACC, middle frontal gyrus, superior and inferior temporal gyrus, and fusiform gyrus. Similarly, other fMRI studies have also reported increased activation in prefrontal regions during similar conditions, in areas that play a key role in cognitive control^16, 20–22^. However, other brain regions have been separately reported to activate during such processes of emotional interference on cognitive control, including the precuneus^17^, insula^23^, precentral gyrus^24^, and postcentral gyrus^25^. Importantly, we believe that some of the inconsistent findings found across the different studies investigating the mechanisms of the emotional Stroop task may not only be related to different subject samples, experimental parameters, or materials used, but also to the degree of emotional interference difficulty used in the task.

Three different types of the emotional Stroop task have been used in neuroimaging studies. Type one is the traditional emotional “color-word” Stroop task, in which participants are asked to name the ink color of words, or count the number of words that are either emotionally-salient or neutral^17, 26, 27^. Longer reaction times for identifying the color of emotional words as compared to neutral words, are regarded as a measure of emotional interference on cognitive control. However, in this traditional variant of the task, the emotional word stimuli are not semantically-relevant to the task instructions^28^ (e.g., naming the ink color of words, or counting the number of words), which results in only mild emotional interference, as previously shown in healthy subjects^6, 29^. A second emotional Stroop task type is the emotional “word-face” task, in which negative or positive words are overlaid on negative or positive facial expressions^6, 21, 30^. The words are either incongruent or congruent with the emotion expressed by the face stimuli, and participants are asked to identify the emotional expression of the faces while ignoring the overlaid emotionally-charged words or vice versa. Facial expressions that differ from the word’s emotional valence (e.g., the word “happy” with an “angry” face) are treated as incongruent conditions. Thus, in contrast to the traditional emotional color-word Stroop task, a semantic conflict is created in this second emotional Stroop task type, resulting in more intense emotional interference^6^ as it requires more effort to complete. A third type of the emotional Stroop task is the “priming” task, in which an emotional or neutral picture is presented prior to “classic Stroop task” trials (e.g., counting Stroop, or color-word Stroop). This task can be used to investigate the influence of emotional priming on cognitive conflict^18, 20^. Similarly to the second type, this type also leads to emotional interference in addition to semantic conflict, thus requiring more effort than the classic Stroop task^18, 20^. In sum, while ‘type one’ emotional Stroop tasks involve mild emotional interference, ‘type two’ and ‘type three’ tasks can create more intense emotional conflict. However, it is unclear whether different brain networks are preferentially involved in intense versus mild emotional interference of cognitive control in emotional Stroop paradigms.

In the current study, we used activation likelihood estimation (ALE) analysis^31^ to quantitatively integrate activation of brain areas reported across different studies that use the emotional Stroop task. ALE is a coordinate-based meta-analysis method that identifies brain areas in which reported foci of activation converge across different experiments^31–33^. This generally involves hundreds of participants and numerous implementations of a specific paradigm or protocol^33–35^. Here we aimed to: (1) recognize a consistent activation pattern of brain regions underlying emotional interference on cognitive control; (2) identify differences in brain activation underlying differing degrees of emotional conflict (mild versus intense); and (3), assess whether the findings provide empirical evidence supporting current theories of emotion-cognition integration^7^.

## Results

### Brain activation underlying emotional interference on cognitive control

**Figure 1:**
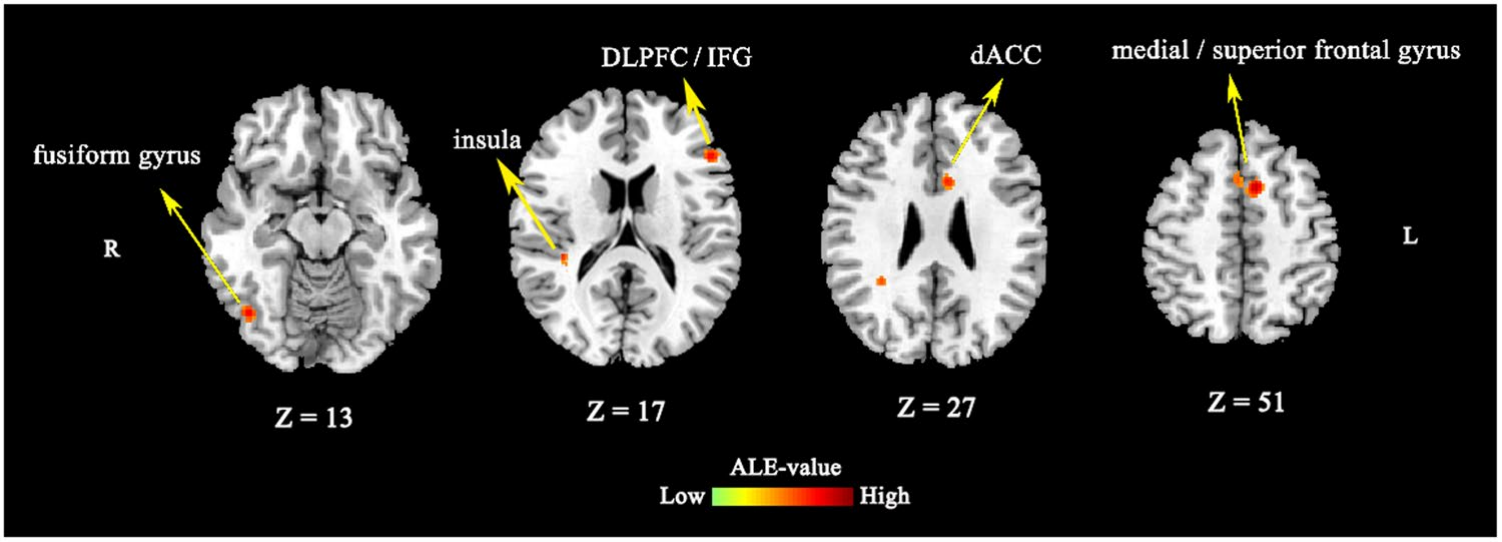
Brain activation underlying emotional interference of cognitive control in emotional Stroop task fMRI studies. DLPFC: dorsolateral prefrontal cortex; IFG: inferior frontal gyrus; dACC: dorsal anterior cingulate cortex. R: right side of the brain; L: left side of the brain. Clusters were displayed using a threshold at *p* < 0.05 (cluster-level, FWE-corrected).

The results from the ALE analysis across all emotional Stroop tasks demonstrated concordance in six main clusters (Table 1; Fig. 1): (1) left medial/superior frontal gyrus (BA6); (2) right medial/superior frontal gyrus (BA32/6); (3) right insula (BA13); (4) left DLPFC/inferior frontal gyrus (BA45/46); (5) right fusiform gyrus (BA19); and (6), left dorsal anterior cingulate cortex (dACC) (BA24).

**Table 1:** Brain activation underlying emotional interference on cognitive control in 16 fMRI studies using an emotional Stroop task.

Cluster	Side	BA	Brain Region	Vol (mm^3^)	Peak Foci (MNI)			ALE (×10^−3^)
					x	y	z	
#1	L	6	Medial/superior frontal gyrus	992	−8	12	54	19.2
#2	R	32/6	Medial/superior frontal gyrus	632	6	14	48	12.9
#3	R	13	Insula	576	34	−46	24	14.4
#4	L	46/45	DLPFC/inferior frontal gyrus	480	−50	30	18	13.9
#5	R	19	Fusiform gyrus	408	44	−68	−10	14.3
#6	L	24	dACC	336	−6	14	26	13.7

DLPFC: dorsolateral prefrontal cortex; dACC: dorsal anterior cingulate cortex.

### Degree of emotional interference difficulty: Intense vs. mild interference

**Figure 2:**
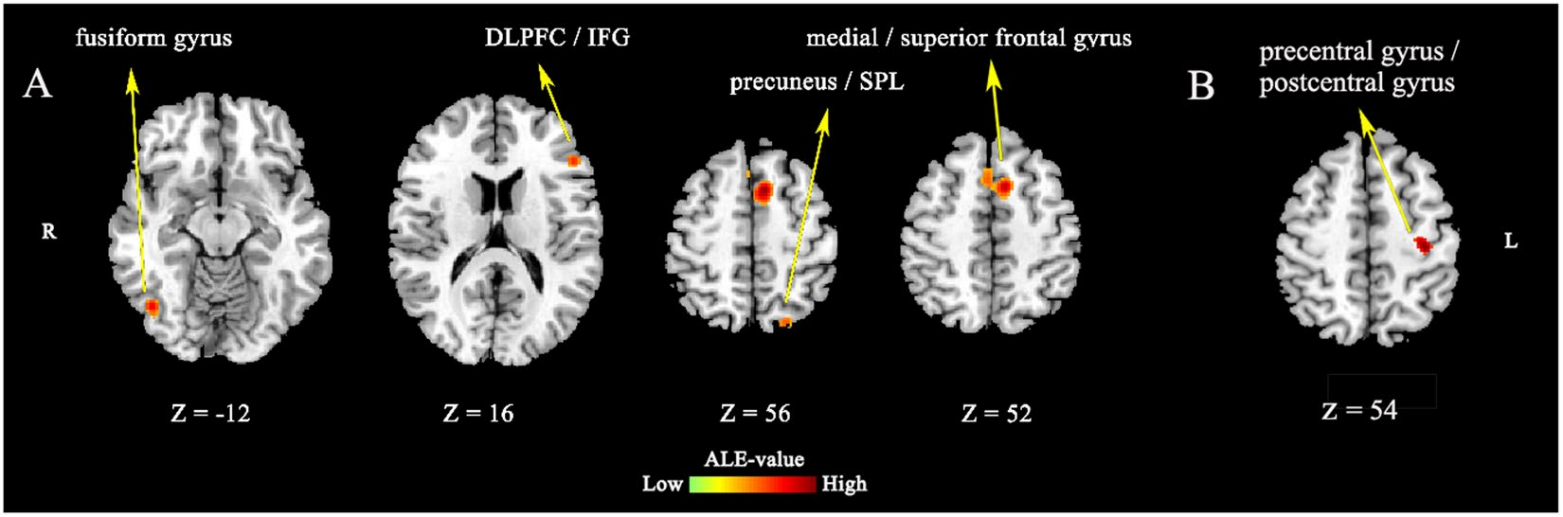
(**A**) Brain activation in emotional Stroop tasks with intense emotional interference. (**B**) Brain activation in emotional Stroop tasks with mild emotional interference. DLPFC: dorsolateral prefrontal cortex; IFG: inferior frontal gyrus; dACC: dorsal anterior cingulate cortex; SPL: superior parietal lobule. R: right side of the brain; L: left side of the brain. Clusters were displayed using a threshold at *p* < 0.05 (cluster-level, FWE-corrected).

A sub-analysis was conducted to assess the influence of conflict degree in the emotional Stroop task. For tasks with *intense* conflict content (N = 9 studies, 60 foci), the ALE analysis demonstrated concurrence in four main clusters (Table 2; Fig. 2A): (1) left medial/superior frontal gyrus (BA6); right medial frontal gyrus (BA6), extending to the dACC; (2) right fusiform gyrus (BA19); (3) left DLPFC/inferior frontal gyrus (BA46/45); and (4), left precuneus/superior parietal lobule (BA7). For tasks with *mild* conflict content (N = 7 studies, 71 foci), concurrent activation was only found in left precentral gyrus (BA4), extending to the postcentral gyrus (BA3) (Table 2; Fig. 2B).

**Table 2:** Brain activation underlying emotional interference on cognitive control during intense versus mild emotional interference.

Cluster	Side	BA	Brain Region	Vol (mm^3^)	Peak Foci (MNI)			ALE (×10^−3^)
					x	y	z	
**Tasks with intense emotional interference** (**9 experiments**)								
#1	L	6	Medial/superior frontal gyrus,	2384	−8	12	54	19.2
	R	6	Medial frontal gyrus, extending to dACC		6	14	48	12.9
#2	R	19	Fusiform gyrus	576	44	−68	−10	14.3
#3	L	46	DLPFC/inferior frontal gyrus	464	−48	30	18	13.2
#4	L	7	Precuneus/SPL	280	−22	−74	58	11.9
**Tasks with mild emotional interference** (**7 experiments**)								
#1	L	4/3	Precentral/postcentral gyrus	392	−34	−24	54	12.5

dACC: dorsal anterior cingulate cortex; DLPFC: dorsolateral prefrontal cortex; SPL: superior parietal lobule.

## Discussion

To the best of our knowledge, this is the first ALE meta-analysis investigating the influence of emotional interference on cognitive control, as specifically measured by the emotional Stroop task. In the current study, we found a consistent pattern of brain activation related to the interaction between the emotional context and cognitive control conditions of the emotional Stroop task, consisting of the medial/superior frontal gyrus, the insula, the DLPFC/IFG, the fusiform gyrus, and the dACC. Furthermore, our sub-analysis showed that tasks with more intense emotional interference consistently elicited increased brain activity in the medial/superior frontal gyrus, the dACC, the fusiform gyrus, the DLPFC/IFG, and the precuneus. In contrast, in tasks with mild emotional conflict, coherent increased brain activation was only found in the precentral/postcentral gyrus. Overall, the coherent activation patterns found in our study are consistent with the theory that cognitive and emotional systems may functionally integrate, sharing underlying neurophysiological mechanisms^7^. Thus, successful engagement of brain areas recruited for executive control may depend on the efficiency of parallel processing for emotional stimuli. Nevertheless, the orchestration between cognitive and emotional systems also likely depends on the nature of the executive task being completed, as well as on the difficulty and complexity of the task, such as the varying degrees of difficulty found across emotional Stroop tasks.

These findings suggest that the resolution of cognitive conflict due to emotional interference on cognitive control is facilitated through a brain network comprising various prefrontal regions. In classic cognitive Stroop experiments without an emotional component, lateral prefrontal activation has been attributed to execution of cognitive control^36–38^, and the dmPFC to activation during conflict detection and monitoring^37, 39, 40^. Similarly however, adjusting one’s response to emotional stimuli requires cognitive control mechanisms that also recruit lateral prefrontal (areas 6/8, 9, 46) and medial frontal regions^41–43^. For example, studies investigating emotional cognitive reappraisal suggest that the dmPFC can be attributed to self-reflective and semantic processes relevant for identifying the emotional value of stimuli^44–46^.

Here we identified lateral PFC activation during emotional interference on cognitive control, particularly within the left DLPFC and left IFG. Previous studies show that the DLPFC is a region that may potentially integrate cognition and emotion, a process needed for the maintenance and updating of emotional information in working memory tasks^47, 48^ and during response inhibition following negative words^49^. A meta-analysis based on the classic Stroop task suggests that the left DLPFC mediates resolution of stimulus conflict, via selective attentional mechanisms^3^. In addition, increased activation of the left IFG in the presence of emotionally-salient stimuli in the present study could argue for a mechanism that is related with top-down suppression of emotional information, a process that may help prioritize the executive task at hand^50^.

Significant activation underlying the influence of emotional interference on cognitive control was also found in the dACC. As a cognitive subdivision of the ACC, the increased dACC activity seen here may represent an essential mechanism for conflict detection^37, 51^, and for adjusting the ongoing need of cognitive control resources^52^. For example, a functional connectivity study has found that connectivity of the dACC predicted DLPFC activation during cognitive conflict trials^53^. Furthermore, studies suggest that the anterior mid-cingulate cortex, anatomically a part of the dACC, may be a potential brain region for the integration of cognitive control and negative emotion^9^.

The insula is involved in a variety of cognitive, emotional, and regulatory functions, and it plays an essential role in facilitating access to attention and working memory^54^. The insula and ACC share an important role in recognizing critical stimuli from sensory input^54^, and activation of this region has been found across different emotion-regulation paradigms (for example in anger, fear and happiness)^55^. The insula is also connected with regions involved in autonomic regulation^56^. Hence, the consistent insular activation in the current study may be a neural correlate of the autonomic changes associated with the subjective experience of emotionally-salient stimuli. Overall, after a stimulus is detected, the insula may support task-related information processing in the fronto-parietal attentional network for task completion purposes. Several studies suggest that recurrent activity in the insula and mid-cingulate cortex may be related to attention-refocusing processes in attention-related tasks^57–59^. Lastly, fusiform gyrus activation in the present analysis falls in line with previous studies showing an activation of this region during shifting of attention towards task-related information/stimuli^60, 61^.

Here we also carried out a sub-analysis comparing intense versus mild, levels of emotional interference difficulty within the emotional Stroop task. In addition to activation of the areas mentioned above, we also found precuneus/superior parietal lobule activation in emotional Stroop tasks with intense emotional conflict. The precuneus (BA7) has been previously shown to be involved in self-referential processing and episodic memory^62^, and the posterior parietal cortex has been previously linked to selective attention, a process where input is filtered to a subset of information and is selected for preferential processing^63^. Additional recruitment of this region may thus occur when emotional interference is more intense, reflecting higher task demands during semantic cognitive conflict trials that require greater attention in order to counteract the distracting effect of the emotionally-salient stimuli^64^. Contrary to paradigms provoking intense conflict however, emotional Stroop tasks provoking only mild emotional conflict showed consistent activation only in the precentral and postcentral gyrus, areas primarily involved in motor behavior^65^ and primary somatosensory sensory input, respectively. When adopting an analysis without multiple comparisons correction *p* < 0.001, the IFG was also part of this activation pattern, a region that as mentioned above may be required for top-down suppression of distracting information.

The above results suggest that the division of the emotional Stroop tasks into subgroups was appropriate, as the emotional Stroop tasks can provoke varying intensities of emotional interference. Thus, future studies exploring similar questions in healthy adults should also consider the differences between these emotional Stroop subtypes.

Contrary to a previous meta-analysis of 43 studies by Cromheeke^11^, we found that the interaction between emotional interference and cognitive control in the current set of studies did not consistently recruit the amygdala or subgenual ACC, the latter of which is considered to be the emotional subdivision of the ACC. There are several explanations for these differences. First, the current study focused only on the emotional Stroop paradigm, while the study of Cromheeke and colleagues, included a broader array of tasks, and thus may have been more prone to finding more brain regions than the present meta-analysis did. Another possibility is that amygdala and subgenual ACC recruitment is observed only in a subset of studies, and that such studies were more heavily sampled in the Cromheeke meta-analysis than in the present one. In fact, in the present meta-analysis, no study reported activation of the amygdala, and only two reported rostral ACC activation. Although the amygdala and subgenual ACC play an important role in processing affect, they may not be entirely critical for the resolution of emotional interference on cognitive control. In fact, these regions may be more likely recruited in paradigms that rely more heavily on holding emotional information in mind (e.g., working memory tasks)^66–68^, resulting in a higher emotional-arousal response. Another possible explanation is that in comparison with the other tasks included in the Cromheeke study, the emotional Stroop task requires more cognitive control for ameliorating the impact of emotion, a process that may preferentially activate the dACC over the subgenual ACC.

Despite the novel results of the current study, there are several limitations to be acknowledged. First, only 16 neuroimaging studies were included in this meta-analysis, limiting the power to detect a common neural mechanism for the emotional interference on cognitive control within emotional Stroop tasks. This was particularly limiting for the subgroup analysis (intense emotional interference subgroup: 9 papers; mild emotional interference subgroup: 7 papers). Therefore, the results of the current study need further discussion and investigation. Second, other cognitive conflict (e.g., emotional Flanker task) or response inhibition tasks (e.g. emotional go/no-go tasks) could not be included in the current study as they lacked enough fMRI papers. Thus, a comparison of the emotional Stroop with other tasks involving cognitive conflict, along with a discussion of any common activation between each of those tasks, was not possible here. Furthermore, it remains unclear whether emotional Stroop tasks cause greater emotional interference in patient populations^16, 19, 22^, as compared to healthy individuals. Lastly, future work is needed to clarify theoretical views on integrative processing. For example, future studies should explore the role of brain areas traditionally thought to be involved in the processing of cognitive conflict, which we are now beginning to see differently because of their additional role in emotional interference resolution.

To conclude, the current study identified a consistent brain activation pattern across an increasing number of studies investigating the influence of emotional interference on cognitive control via the emotional Stroop paradigm. Specifically, regions commonly thought to be involved in cognitive control (e.g., DLPFC, IFG, dACC), along with the insula, showed increased activation during the performance of a cognitive control task with emotional interference. Importantly however, these activations were heavily influenced by emotional conflict level.

## Methods

### Literature search

To identify pertinent articles, a systematic database search of the Web of Science and PubMed databases was performed for peer-reviewed articles published between January 1990 and January 2016. Search terms for the emotional Stroop task were combined with different fMRI-related terms. The search keywords related to emotional Stroop that were used were “Stroop”, coupled with one or two of the search terms below: “cognitive control”, “cognitive interference”, “affective”, “emotion”, “emotional interference”, “cognition–emotion”, “emotion–cognition”. The search keywords related to fMRI used were “fMRI, functional magnetic resonance imaging, functional imaging, neuroimaging, functional MRI, functional magnetic imaging”.

### Inclusion and exclusion criteria

**Figure 3:**
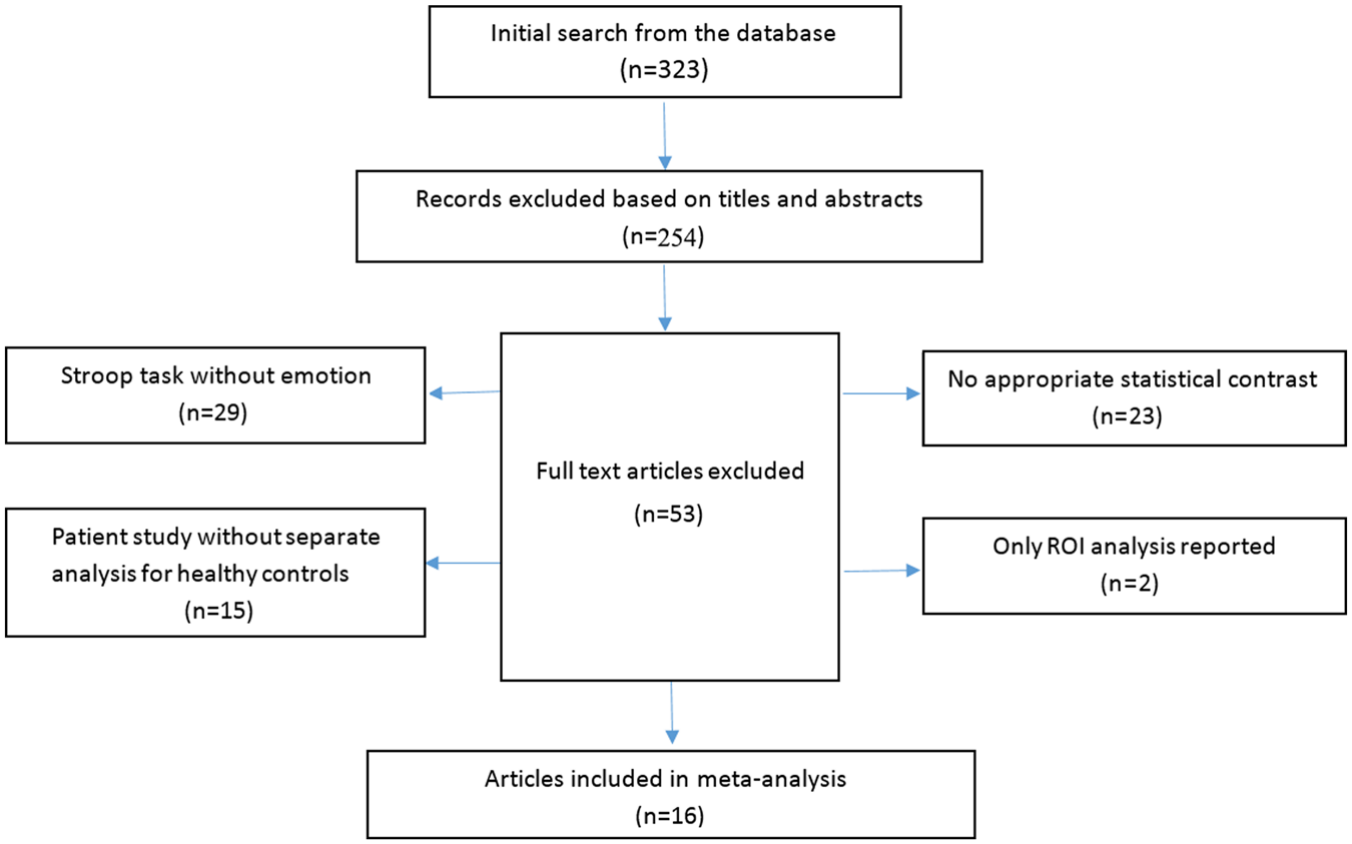
Flowchart of the study selection process.

For inclusion, the research studies were required to include an emotional Stroop task in healthy adults. Furthermore, as we intended to explore the neural correlates of the interaction between emotional interference and cognitive processing, all the fMRI studies included report three-dimensional Talairach or Montreal Neurologic Institute (MNI) coordinates for interaction effects between the emotional manipulation and cognitive control. Studies were excluded (Fig. 3) if: (1) the Stroop task lacked an emotional context (i.e., Stroop task studies were excluded if they did not contain emotion-related materials or stimuli); (2) the study that did not report an interaction between the emotional manipulation and cognitive control (i.e., there was no appropriate statistical contrast), (for more details, please see “contrast selection” section); (3) the study included patients and had no separate within-group analysis for healthy controls; or (4) the study conducted a region of interest analysis based on previous research, but not a whole-brain analysis. The final meta-analysis included a total of 16 eligible fMRI studies using an emotional Stroop task, and a total of 330 participants, equaling 132 foci (Table 3). 10 studies of them were cross-sampled from Cromheeke’s meta-analysis paper^11^, and in addition, 6 new papers were also included in the current study. Foci that were located outside the Ginger ALE 2.3.5 gray matter mask were excluded from all analyses.

**Table 3:** Characteristics of studies included in meta-analysis.

Study	N	Emotional stimuli	Experimental contrast	Task type	Task with intense or mild conflict
Malhi *et al*., 2005	12	negative, positive, neutral words	Emotional Stroop (negative + positive) > neutral Stroop	Type one	mild
Mohanty *et al*., 2005	17	positive, negative, neutral words	Negative Stroop > neutral Stroop	Type one	mild
Blair *et al*., 2007	22	IAPS (negative, positive, neutral)	Negative (incongruent vs. baseline) > neutral (incongruent vs. baseline); positive (incongruent vs. baseline) > neutral (incongruent vs. baseline)	Type three	intense
Mitterschiffthaler *et al*., 2007	17	sad, neutral words	Negative Stroop > neutral Stroop	Type one	mild
Park *et al*., 2008	14	positive, negative	Emotional incongruence > emotional congruence	Type two	intense
Wingenfeld *et al*., 2009	20	neutral, general negative and individual negative words	Negative Stroop > neutral Stroop	Type one	mild
Chechko *et al*., 2009	18	happy, fearful faces	Emotional incongruence > emotional congruence (in controls);	Type two	intense
Hart *et al*., 2010	14	IAPS (aversive, neutral)	Aversive incongruent > neutral incongruent	Type three	intense
Melcher *et al*., 2011	14	IAPS (negative, neutral) faces	Negative incongruent versus baseline	Type three	intense
Chechko *et al*., 2012	24	happy, sad, fearful	Emotional (incongruent > congruent) -non-emotional (incongruent > congruent)	Type two	intense
Chechko *et al*., 2013	18	happy, sad, fearful	Emotional (incongruent > congruent) -non-emotional (incongruent > congruent)	Type two	intense
Rahm *et al*., 2013	11	sad, Fearful, Neutral words	Emotional negative Stroop > Emotional neutral Stroop	Type one	mild
Veroude *et al*., 2013	74	positive, negative, neutral words	Negative Stroop > Neutral Stroop	Type one	mild
Han *et al*., 2014	14	positive, negative, neutral scenes	Negative (incongruent > congruent); Neutral (incongruent > congruent); Positive (incongruent > congruent)	Type three	intense
Rey *et al*., 2014	12	fearful, joyful	Emotional (incongruent > congruent)	Type two	intense
Brennan *et al*., 2015	29	negative, neutral	Negative Stroop > Neutral Stroop	Type one	mild

N: number of participants. Type one: “color-word” emotional Stroop task; type two: “word-face” emotional Stroop task; type three: “priming” emotional Stroop task.

### Contrast selection

The terms “incongruent” and “congruent” for the emotional Stroop task were defined as follows: (1) for ‘type one’ Stroop tasks (traditional emotional “color-word” Stroop tasks)^17, 26, 27^, emotional word stimuli were considered to be the “incongruent” condition, as emotional stimuli potentially distract from the main task goal (e.g., to count the number of words, or identify the color of the word). Correspondingly, the emotionally neutral words were considered as the “congruent” condition. (2) For ‘type two’ Stroop tasks (emotional “word-face” Stroop tasks)^6, 21, 30^, the condition in which the emotional facial expression differed from the overlaid word’s emotional valence (e.g., the word “happy” with an “angry” face) was treated as the “incongruent” condition, while trials in which the facial expression was similar to the word’s emotional valence (e.g., the word “happy” with a “happy” face) were treated as the “congruent” condition. (3) For ‘type three’ Stroop tasks (“priming” emotional Stroop tasks)^18, 20^, the condition in which the color of the word differed from its lexical meaning (e.g., the word “blue” in red color) was treated as the “incongruent” condition, while trials with the color fitting its lexical meaning (e.g., the word “blue” in blue color) were treated as the “congruent” condition.

Because this study sought to examine the influence of emotional interference on cognitive control as measured by the emotional Stroop task, we only selected studies that reported an interaction between emotional context and cognitive control conditions. In such paradigms, “emotional conflict”, or “emotional interference”, were measured by using emotional stimuli in one of these contrasts: “incongruent vs. congruent”, “incongruent vs. baseline”, or just “incongruent” (e.g., “emotional incongruent vs. neutral incongruent”). Thus, the selected statistical contrasts for inclusion in the current study were (for information on specific studies, please see Table 3): (1) Emotional (incongruent > congruent) - non-emotional (incongruent > congruent); (2) Emotional incongruent > neutral incongruent; (3) Emotionally incongruent > emotionally congruent; or (4) Emotional Stroop > neutral Stroop.

As few studies (only three) reported deactivations that met our criteria, these results could not be used to calculate effective ALE metrics. Thus, only studies reporting increased activation were included in the current study^11^. In summary, we first conducted a meta-analysis to explore consistent brain activation across all emotional Stroop tasks, and then performed a sub-analysis to explore the influence of emotional conflict level (intense versus mild interference) on cognitive control.

### Activation likelihood estimation

For each meta-analysis, we adopted the standard ALE meta-analytic approach^31–33^ (http://brainmap.org/ale). As ALE was performed in MNI stereotactic space, all coordinates reported in Talairach coordinates were transformed into MNI locations before ALE analysis^69^. In an ALE analysis, the first step is modeling single study activation foci as peaks of three-dimensional Gaussian probability densities with full-width at half-maximum (FWHM) values based on the number of participants^33^; then, in order to produce a statistical map estimating the likelihood of activation at each voxel and calculate the summation of probability densities, the ALE map is tested based on an ALE null distribution map resulting from a permutation procedure to determine statistical significance^34, 70^; lastly, correction for multiple comparisons based on a permutation test is performed to acquire a thresholding of the maximum cluster size needed for statistical significance. For each meta-analysis, we used a family-wise error-correction (FWE) at the cluster level threshold of *p* < 0.05 (cluster-forming threshold at voxel-level *p* < 0.001, 5000 permutations). The resulting thresholded ALE images were overlaid onto an anatomical T1-weighted image in MNI space.

## References

[1] Miyake A (2000). The unity and diversity of executive functions and their contributions to complex “frontal lobe” tasks: A latent variable analysis. Cognitive psychology.

[2] Banich MT (2009). Cognitive control mechanisms, emotion and memory: a neural perspective with implications for psychopathology. Neuroscience & Biobehavioral Reviews.

[3] Nee DE, Wager TD, Jonides J (2007). Interference resolution: insights from a meta-analysis of neuroimaging tasks. Cognitive, Affective, & Behavioral Neuroscience.

[4] LeDoux JE (2000). Emotion Circuits in the Brain. Annual Review of Neuroscience.

[5] Mathews A (1990). Why worry? The cognitive function of anxiety. Behaviour research and therapy.

[6] Etkin A, Egner T, Peraza DM, Kandel ER, Hirsch J (2006). Resolving emotional conflict: a role for the rostral anterior cingulate cortex in modulating activity in the amygdala. Neuron.

[7] Pessoa L (2008). On the relationship between emotion and cognition. Nature Reviews Neuroscience.

[8] Mueller S (2011). The influence of emotion on cognitive control: relevance for development and adolescent psychopathology. Frontiers in psychology.

[9] Shackman AJ (2011). The integration of negative affect, pain and cognitive control in the cingulate cortex. Nature reviews. Neuroscience.

[10] Okon-Singer H, Hendler T, Pessoa L, Shackman AJ (2015). The neurobiology of emotion-cognition interactions: fundamental questions and strategies for future research. Front Hum Neurosci.

[11] Cromheeke S, Mueller SC (2014). Probing emotional influences on cognitive control: an ALE meta-analysis of cognition emotion interactions. Brain Structure and Function.

[12] Swick D, Ashley V, Turken U (2011). Are the neural correlates of stopping and not going identical? Quantitative meta-analysis of two response inhibition tasks. NeuroImage.

[13] Eriksen BA, Eriksen CW (1974). Effects of noise letters upon the identification of a target letter in a nonsearch task. Perception & psychophysics.

[14] Ochsner KN, Hughes B, Robertson ER, Cooper JC, Gabrieli JD (2009). Neural systems supporting the control of affective and cognitive conflicts. Journal of cognitive neuroscience.

[15] Kanske P, Kotz SA (2011). Emotion triggers executive attention: anterior cingulate cortex and amygdala responses to emotional words in a conflict task. Human brain mapping.

[16] Malhi GS, Lagopoulos J, Sachdev PS, Ivanovski B, Shnier R (2005). An emotional Stroop functional MRI study of euthymic bipolar disorder. Bipolar Disorders.

[17] Rahm C, Liberg B, Wiberg-Kristoffersen M, Aspelin P, Msghina M (2013). Rostro-caudal and dorso-ventral gradients in medial and lateral prefrontal cortex during cognitive control of affective and cognitive interference. Scandinavian journal of psychology.

[18] Melcher T, Born C, Gruber O (2011). How negative affect influences neural control processes underlying the resolution of cognitive interference: an event-related fMRI study. Neuroscience research.

[19] Mohanty A (2005). Neural mechanisms of affective interference in schizotypy. Journal of abnormal psychology.

[20] Hart SJ, Green SR, Casp M, Belger A (2010). Emotional priming effects during Stroop task performance. NeuroImage.

[21] Rey G (2014). Modulation of brain response to emotional conflict as a function of current mood in bipolar disorder: preliminary findings from a follow-up state-based fMRI study. Psychiatry research.

[22] Mitterschiffthaler M (2008). Neural basis of the emotional Stroop interference effect in major depression. Psychological medicine.

[23] Chechko N (2013). Brain circuitries involved in emotional interference task in major depression disorder. J Affect Disord.

[24] Chechko N, Kellermann T, Zvyagintsev M, Augustin M, Schneider F (2012). Brain circuitries involved in semantic interference by demands of emotional and non-emotional distractors. PloS one.

[25] Veroude K, Jolles J, Croiset G, Krabbendam L (2013). Changes in neural mechanisms of cognitive control during the transition from late adolescence to young adulthood. Developmental cognitive neuroscience.

[26] Mathews A, MacLeod C (1985). Selective processing of threat cues in anxiety states. Behaviour research and therapy.

[27] Wingenfeld K (2009). Neural correlates of the individual emotional Stroop in borderline personality disorder. Psychoneuroendocrinology.

[28] Whalen PJ, Bush G, Shin LM, Rauch SL (2006). The emotional counting Stroop: a task for assessing emotional interference during brain imaging. Nature Protocols-Electronic Edition-.

[29] Williams JMG, Mathews A, MacLeod C (1996). The emotional Stroop task and psychopathology. Psychological bulletin.

[30] Chechko N (2009). Unstable prefrontal response to emotional conflict and activation of lower limbic structures and brainstem in remitted panic disorder. PLoS One.

[31] Turkeltaub PE, Eden GF, Jones KM, Zeffiro TA (2002). Meta-analysis of the functional neuroanatomy of single-word reading: method and validation. NeuroImage.

[32] Laird AR (2005). ALE meta-analysis: Controlling the false discovery rate and performing statistical contrasts. Human brain mapping.

[33] Eickhoff SB (2009). Coordinate-based activation likelihood estimation meta-analysis of neuroimaging data: A random-effects approach based on empirical estimates of spatial uncertainty. Human brain mapping.

[34] Laird AR (2009). Investigating the functional heterogeneity of the default mode network using coordinate-based meta-analytic modeling. The Journal of Neuroscience.

[35] Eickhoff SB, Bzdok D, Laird AR, Kurth F, Fox PT (2012). Activation likelihood estimation meta-analysis revisited. NeuroImage.

[36] Egner T, Hirsch J (2005). The neural correlates and functional integration of cognitive control in a Stroop task. NeuroImage.

[37] Carter CS, Van Veen V (2007). Anterior cingulate cortex and conflict detection: an update of theory and data. Cognitive, Affective, & Behavioral Neuroscience.

[38] Egner T, Etkin A, Gale S, Hirsch J (2008). Dissociable neural systems resolve conflict from emotional versus nonemotional distracters. Cerebral cortex.

[39] MacDonald AW, Cohen JD, Stenger VA, Carter CS (2000). Dissociating the role of the dorsolateral prefrontal and anterior cingulate cortex in cognitive control. Science.

[40] Botvinick MM, Cohen JD, Carter CS (2004). Conflict monitoring and anterior cingulate cortex. Trends in Cognitive Sciences.

[41] Beauregard, M., Levesque, J. & Bourgouin, P. Neural correlates of conscious self-regulation of emotion. *The Journal of neuroscience* (2001). 10.1523/JNEUROSCI.21-18-j0001.2001 PMC6763007 11549754

[42] Ochsner KN, Bunge SA, Gross JJ, Gabrieli JD (2002). Rethinking feelings: an FMRI study of the cognitive regulation of emotion. Journal of cognitive neuroscience.

[43] Lévesque J (2003). Neural circuitry underlying voluntary suppression of sadness. Biological psychiatry.

[44] Crosson B (2002). Semantic monitoring of words with emotional connotation during fMRI: contribution of anterior left frontal cortex. Journal of the International Neuropsychological Society.

[45] Amodio DM, Frith CD (2006). Meeting of minds: the medial frontal cortex and social cognition. Nature Reviews Neuroscience.

[46] Olsson A, Ochsner KN (2008). The role of social cognition in emotion. Trends in cognitive sciences.

[47] Perlstein WM, Elbert T, Stenger VA (2002). Dissociation in human prefrontal cortex of affective influences on working memory-related activity. Proceedings of the National Academy of Sciences.

[48] Gray JR, Braver TS, Raichle ME (2002). Integration of emotion and cognition in the lateral prefrontal cortex. Proceedings of the National Academy of Sciences.

[49] Goldstein M (2007). Neural substrates of the interaction of emotional stimulus processing and motor inhibitory control: an emotional linguistic go/no-go fMRI study. NeuroImage.

[50] Swick D, Ashley V, Turken U (2008). Left inferior frontal gyrus is critical for response inhibition. BMC neuroscience.

[51] Kerns JG (2004). Anterior cingulate conflict monitoring and adjustments in control. Science.

[52] Botvinick MM, Braver TS, Barch DM, Carter CS, Cohen JD (2001). Conflict monitoring and cognitive control. Psychological review.

[53] Mohanty A (2007). Differential engagement of anterior cingulate cortex subdivisions for cognitive and emotional function. Psychophysiology.

[54] Menon V, Uddin LQ (2010). Saliency, switching, attention and control: a network model of insula function. Brain Structure and Function.

[55] Damasio AR (2000). Subcortical and cortical brain activity during the feeling of self-generated emotions. Nature neuroscience.

[56] Cechetto DF (1994). Identification of a cortical site for stress-induced cardiovascular dysfunction. Integrative physiological and behavioral science.

[57] Dickstein SG, Bannon K, Xavier Castellanos F, Milham MP (2006). The neural correlates of attention deficit hyperactivity disorder: An ALE meta-analysis. Journal of Child Psychology and Psychiatry.

[58] Sridharan D, Levitin DJ, Menon V (2008). A critical role for the right fronto-insular cortex in switching between central-executive and default-mode networks. Proceedings of the National Academy of Sciences.

[59] Fischer T, Langner R, Diers K, Brocke B, Birbaumer N (2010). Temporo-spatial dynamics of event-related EEG beta activity during the initial contingent negative variation. PLoS One.

[60] Vuilleumier P, Armony JL, Driver J, Dolan RJ (2001). Effects of attention and emotion on face processing in the human brain: an event-related fMRI study. Neuron.

[61] Mather M (2006). Emotional arousal can impair feature binding in working memory. Journal of cognitive neuroscience.

[62] Cavanna AE, Trimble MR (2006). The precuneus: a review of its functional anatomy and behavioural correlates. Brain.

[63] Behrmann M, Geng JJ, Shomstein S (2004). Parietal cortex and attention. Current opinion in neurobiology.

[64] Wessa, M., Heissler, J., Schönfelder, S. & Kanske, P. Goal-directed behavior under emotional distraction is preserved by enhanced task-specific activation. *Social cognitive and affective neuroscience* nsr098 (2012). 10.1093/scan/nsr098 PMC3594722 22302842

[65] Sanes JN, Donoghue JP (2000). Plasticity and primary motor cortex. Annual review of neuroscience.

[66] Dolcos F, McCarthy G (2006). Brain systems mediating cognitive interference by emotional distraction. The Journal of Neuroscience.

[67] Habel U (2007). The influence of olfactory-induced negative emotion on verbal working memory: individual differences in neurobehavioral findings. Brain research.

[68] Dolcos F, Diaz-Granados P, Wang L, McCarthy G (2008). Opposing influences of emotional and non-emotional distracters upon sustained prefrontal cortex activity during a delayed-response working memory task. Neuropsychologia.

[69] Lancaster JL (2007). Bias between MNI and Talairach coordinates analyzed using the ICBM-152 brain template. Human brain mapping.

[70] Caspers S, Zilles K, Laird AR, Eickhoff SB (2010). ALE meta-analysis of action observation and imitation in the human brain. NeuroImage.

